# Fluorescence Spectroscopy for Studying Evaporating Droplets Using the Dye Eosin-Y

**DOI:** 10.3390/s20215985

**Published:** 2020-10-22

**Authors:** Matthias Koegl, Christoph Weiß, Lars Zigan

**Affiliations:** 1 Lehrstuhl für Technische Thermodynamik (LTT), Friedrich-Alexander-Universität Erlangen-Nürnberg (FAU), 91058 Erlangen, Germany; Christoph1.weiss@tum.de (C.W.); lars.zigan@fau.de (L.Z.); 2 Erlangen Graduate School in Advanced Optical Technologies (SAOT), Friedrich-Alexander-Universität Erlangen-Nürnberg (FAU), 91052 Erlangen, Germany

**Keywords:** absorption, LIF, ethanol, water, dye, acoustic levitator

## Abstract

Laser-induced fluorescence (LIF) spectroscopy using dyes is frequently applied for characterization of liquids and two-phase flows. The technique is utilized e.g., for mixing studies, thermometry, or droplet sizing. One major application of the LIF technique combined with Mie-scattering is the planar measurement of droplet sizes in spray systems. However, its uncertainty is determined, among others, by varying dye concentration and temperature changes occurring during mixing and droplet evaporation. Systematic experimental investigations are necessary to determine the influence of dye enrichment effects on the LIF-signal of single droplets. For these investigations, the fluorescence dye Eosin-Y is dissolved in water and ethanol, which are typical solvents and working fluids in bio-medical applications and power engineering. A photo-physical characterization of the mixtures under various conditions was conducted using a spectrometric LIF setup and a micro cell. For ethanol, a small temperature dependency of the Eosin-Y LIF signal is observed up to 373 K. Photo-dissociation of Eosin-Y is negligible for solution in ethanol while it is distinct in water. The LIF signals of the single droplets are studied with an acoustic levitator. Effects of droplet evaporation, droplet deformation and varying dye concentration on the LIF-signal are studied. The single droplet measurements revealed a complex change of the fluorescence signal with reduced droplet size. This is due to droplet deformations leading to variations in the internal illumination field as well as dye enrichment during evaporation.

## 1. Introduction

The knowledge of the droplet characteristics, especially the droplet size distribution, is essential for the improvement of sprays in bio-medical, pharmaceutical, and automotive applications as well as for sprays in the food industry and agriculture [1,2,3]. The prediction of atomization and evaporation characteristics using semi-empirical models in CFD (CFD- computational fluid dynamics) is often not accurate enough and additional experimental data is needed for their validation. For this purpose, many non-invasive techniques were developed and improved over the last decades. Laser-induced fluorescence (LIF) is one technique for determination of droplet composition and temperature [4]. Additionally, planar droplet sizing is possible using LIF in combination with Mie-scattering [5,6,7,8,9,10,11,12,13,14]. Besides this LIF/Mie ratio based droplet sizing, other techniques like the Raman/Mie ratio [15] and the LIEF/Mie ratio [16] (LIEF—laser-induced exciplex fluorescence) were developed to determine the droplet size distribution. Especially for dilute spray regions, shadowgraphy [17,18] and interferometric Mie Imaging [19] are appropriate for droplet sizing as well.

The LIF/Mie ratio technique enables the spray characterization in terms of the Sauter mean diameter (SMD). A dye is typically added to a non-fluorescent liquid. Eosin-Y is a suitable dye for ethanol and water, as it shows high fluorescence quantum yield and low temperature sensitivity, which is advantageous for planar droplet sizing (PDS) [20,21]. The technique is based on the assumption, that the LIF-signal of a laser light illuminated droplet is approximately volume dependent (i.e., it is proportional to *d*^3^) and the Mie-scattering signal is surface dependent (i.e., it is proportional to *d*^2^) [5,6,7,8,9,10,11,12,13,14]. Besides these assumptions, previous investigations showed that the LIF exponent is highly absorption dependent. A low absorption (low dye concentration) leads to an exponent of 3 according to the literature, while a high absorption (high dye concentration) leads to a decrease of the LIF-exponent up to 2 [5]. However, there are further effects on the LIF signal leading to complex dependencies, which are not known in detail. Some of them will be discussed below.

The LIF/Mie ratio is initially a qualitative measurement technique. To obtain a quantitative SMD-distribution, an adequate calibration with a droplet generator [8,14] or Phase-Doppler Anemometry (PDA) measurements [5,21] is required. Fundamental work for improving the LIF/Mie technique under evaporating conditions can also be conducted using levitated single droplets. This configuration is advantageous as individual droplets can be studied over a longer time period in which evaporation effects become dominant leading to dye enrichment in the droplets.

The LIF/Mie technique is very sensible towards varying dye concentration during evaporation and temperature changes, which can cause large errors in SMD. Up to now, only a few studies investigated these issues [12,13,22], but no systematic work on the spectrometric properties of the fuel–tracer mixture itself and evaporating droplets were conducted. Especially, data for ethanol sprays in combination with the tracer Eosin-Y [14,21] are missing. Small temperature intervals were investigated to assess the temperature dependence of the fluorescence signal in a cuvette of Eosin-Y dissolved in ethanol (ΔT~30 K) and water (ΔT~40 K) [21,23]. Liquid temperature effects on the LIF/Mie-ratio of monodisperse droplets were analyzed using a droplet generator, however, dye enrichment during evaporation can barely be investigated [14]. Other sources of uncertainty are for example the droplet deformation, which can hardly be controlled in typical droplet (chain) generators [14,24].

In the present study, the influence of dye enrichment in evaporating single droplets doped with Eosin-Y is studied with an acoustic levitator. In the first step, the photo-physical properties of the mixtures are analyzed in a micro cell under various conditions using a spectrometric LIF setup. The present work extended the temperature sensitivity interval of the absorption and emission of Eosin-Y dissolved in ethanol in the literature (296 K–338 K, [20,21]) to 283 K–413 K. Furthermore, the single droplet investigations extend the knowledge regarding the measurement uncertainties of the LIF/Mie droplet sizing technique at ambient conditions and long droplet residence times, leading to a change in dye concentration as well as droplet deformation. These studies provide deeper insights into individual effects on the LIF signal supporting the error estimation of PDS.

## 2. Experimental Setup

### 2.1. Fluorescence Spectroscopy Setup

**Figure 1:**
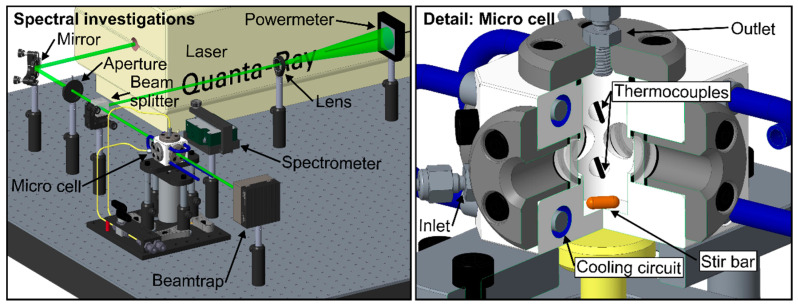
Optical setup (**left**) and internal design (sectional view) of the micro cell (**right**).

The ethanol-eosin and water-eosin mixtures are characterized using a specially designed micro cell. The optical setup and a sectional view of the micro cell are shown in Figure 1. The probe volume within the micro cell is illuminated by a pulsed Nd:YAG laser (model 150-10, Spectra-Physics, Santa Clara, CA, USA; wavelength 532 nm (FWHM: <1 nm), repetition rate 10 Hz, pulse width > 10 ns, laser fluence: 45.8 mJ/cm^2^). The usage of an electrically actuated shutter inside the laser enables a continuous laser operation. The shutter is only opened during the measurements and closed between the operating points. This procedure also avoids possible photo-dissociation effects due to constant probe illumination. The emitted laser beam cross section is cut down to 4.2 mm by an aperture. The beam is then bisected by a beam splitter to enable simultaneous monitoring of the laser fluence (power meter: model QE50LP-S-MB-INT-D0, Gentec Electro-Optics, Québec, QC, Canada) during the measurements. One of the bisected beams passes the measurement volume in the micro cell (which was used for temperature dependent measurements) or a cuvette (used for concentration studies), respectively. A spectrometer (model USB 4000, Ocean Optics, Dunedin, FL, USA, wavelength range 495.9 nm–831.8 nm, 3648 pixels, slit size 10 µm, integration time 100 ms, 50 subsequent spectra were averaged for each measurement) records the LIF-spectra under a detection angle of 90°. For studying the effect of dye concentration and ethanol concentration on the fluorescence signal, which was conducted at ambient conditions (0.1 MPa, 293 K), a cuvette with a quadratic cross section (Hellma, Müllheim, Germany, edge length 10 mm, 3.5 mL, quartz glass SUPRASIL^®^) was used. The temperature-dependent emission spectra were recorded in the micro cell. The micro cell features four optical accesses (½” sapphire windows, optical access diameter: 9 mm, inner distance between two windows: 19.1 mm). A built-in magnetic stirrer (stir bar: 8 mm × 3 mm, 1500 rpm) ensures a homogeneous temperature distribution within the cell. The temperature within the micro cell is monitored by two thermocouples (type K). To avoid effects of photo-dissociation, the investigated mixture inside the cell can be exchanged between the measurements. Cooling/heating circuits in the cell body in combination with a recirculating thermostat (type: Julabo FP50, not shown in the figure) enable a wide range of investigated temperatures. The tracer and fuel concentration dependent absorption measurements were performed using a stand-alone UV/VIS spectrometer (model V-750, JASCO, Japan, not shown in the figure) with a wavelength range 190 nm–900 nm. Its specifications are 3551 pixels, 2 nm spectral bandwidth and a scan speed 200 nm/min.

### 2.2. Imaging Setup for the Droplet Study

**Figure 2:**
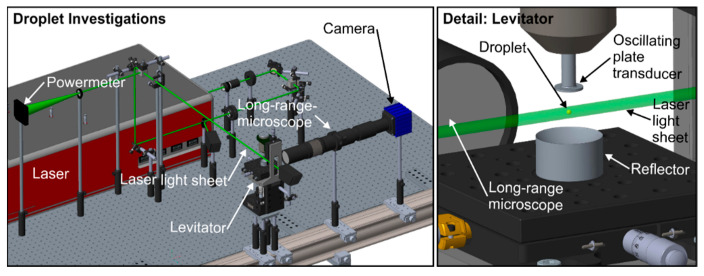
Optical setup (**left**) and detailed view of the levitator (**right**) for the droplet measurements.

The optical setup for the detection of the microscopic LIF signals of micrometric droplets is shown in Figure 2. The levitator (58 KHz, tec5, Oberursel, Germany) is fixed on a 3-dimensional axis system, with the transducer in the roof and the reflector in its base. The presented study deals with macro droplets (initial droplet diameter: 1600 µm), which ensure a simple handling. The droplets (3.5 µL, droplet diameter~1600 µm) were delivered manually through a 5 µL gas chromatography syringe (#7105, Hamilton, Bonaduz, Switzerland) into the central pressure node of the standing wave. Smaller droplets (50 µm–100 µm), which are more representative for automotive and industrial spray systems can only be generated by piezo-actuated dispensers or related devices, which will be part of future work. A pulsed 532 nm laser (Multi-Nd:YAG-Laser, Thales, Paris, France with top-hat beam profile, FWHM: <1 nm, beam-diameter 8 mm, laser fluence: 2.5 mJ/cm^2^) illuminates the droplets. The laser fluence is very low in comparison to previous droplet measurements [14,24]. High laser fluences in combination with high dye concentrations result in very bright spots at the droplet surface leading to an oversaturation of certain camera pixels. An intensity reduction with appropriate neutral density filters or an aperture would lead to low fluorescence intensities inside the droplets.

The beam is bisected by a beam splitter to enable simultaneous monitoring of the laser fluence (power meter: model QE50LP-S-MB-INT-D0, Gentec Electro-Optics, Québec, QC, Canada) during the measurements. The LIF images of the levitated droplets were recorded with a sCMOS camera (Imager, LaVision, Goettingen, Germany; resolution: 2560 pixel × 2160 pixel) and a long-range microscope (Navitar, Rochester, NY, USA) with a nominal spatial resolution of 730 pixel/µm. The Mie-scattering was blocked using a suitable filter (532 nm notch filter, 17 FWHM), which was mounted directly in front of the camera.

### 2.3. Dye and Solvents

Eosin-Y is a solid acid xanthene (natural anionic) dye [25]. Besides widespread application in the field of medicine [26] and biology [27], the fluorescence tracer is used for planar droplet sizing for alcohol and water sprays [14,21,28], and as groundwater migration tracer [29]. To investigate the effects on partial evaporation of droplets on the fluorescence signal, the dye Eosin-Y is dissolved in ethanol and water. The dye was completely dissolved in all investigated fuel–dye mixtures. The solutions were diluted to adjust the respective dye concentrations.

## 3. Results of the Dye Characterization

This section is structured as follows: First, the dye concentration effect on the absorption and fluorescence spectra of ethanol and water is investigated. Second, the influence of the laser power on the fluorescence signal is analyzed. Third, the temperature dependencies of the emission and absorption spectra are investigated. Finally, the effect of photo-dissociation on the fluorescence is analyzed. All spectral results are presented in the visible wavelength range (380 nm–780 nm), which is most relevant for the absorption and emission of Eosin-Y.

### 3.1. Dye Concentration Effect on Absorption and Emission Spectra of Eosin-Y

**Figure 3:**
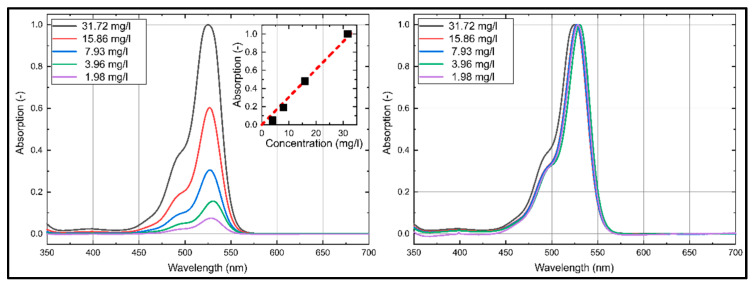
Absorption spectra (**left**: normalized to maximum intensity of 31.724 mg/L; **right**: normalized to the respective maximum intensity) of Eosin-Y dissolved in ethanol, inserted diagram show linearity (*R*^2^ = 0.981) of the integral laser-induced fluorescence (LIF)-signal for various dye concentrations; 293 K, 0.1 MPa.

**Figure 4:**
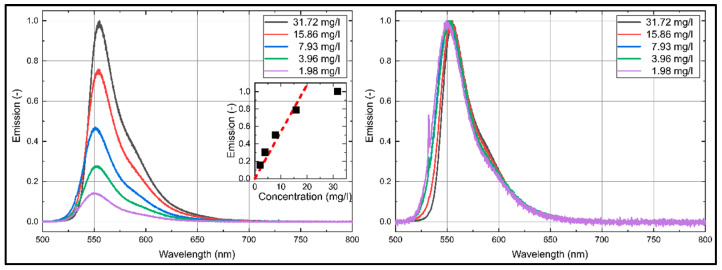
Emission spectra (**left**: normalized to maximum intensity of 31.72 mg/L; **right**: normalized to the respective maximum intensity) of Eosin-Y dissolved in ethanol, inserted diagram show linearity (*R*^2^ = 0.980) of the integral LIF-signal for various dye concentrations; 293 K, 0.1 MPa.

The emission and absorption spectra of Eosin-Y dissolved in ethanol and water at various dye concentrations at reference conditions (293 K, 0.1 MPa) are discussed in the current section. The absorption and emission spectra are presented for the visible wavelength region (350 nm–800 nm) in Figure 3 and Figure 4, which is important for an excitation at 532 nm and a detection in the visible wavelength region.

First, the absorption and emission characteristics of Eosin-Y dissolved in ethanol are discussed. The absorption spectrum of Eosin-Y shows a distinct absorption between 440 nm and 575 nm with a broad peak around 527 nm. The absorption intensities increase linearly with higher dye concentration for all investigated concentrations. The coefficient of determination *R*^2^ for the linear fit curve displayed in Figure 3 is 0.981. An increase in dye concentration slightly shifts the absorption spectra towards lower wavelengths.

The emission spectra of Eosin-Y in ethanol show a distinct emission between 525 nm and 675 nm, see Figure 4. The emission spectra consist of one broad peak around 555 nm. The emission intensities increase linearly with higher dye concentration up to 15.86 mg/L. Dye concentrations above 15.86 mg/L lead to a saturation of the fluorescence caused by extinction effects. The coefficient of determination *R*^2^ for the linear fit curve (up to 15.86 mg/L) displayed in Figure 4 is 0.980. An increase in dye concentration of Eosin-Y in ethanol slightly shifts the emission spectra towards higher wavelengths, which is better visible in the normalized emission spectra in Figure 4 (right).

**Figure 5:**
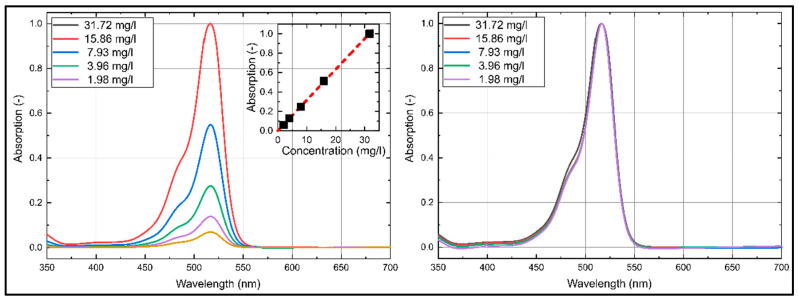
Absorption spectra (**left**: normalized to maximum intensity of 31.72 mg/L; **right**: normalized to the respective maximum intensity) of Eosin-Y dissolved in water, inserted diagram show linearity (*R*^2^ = 0.999) of the integral LIF-signal for various dye concentrations; 293 K, 0.1 MPa.

**Figure 6:**
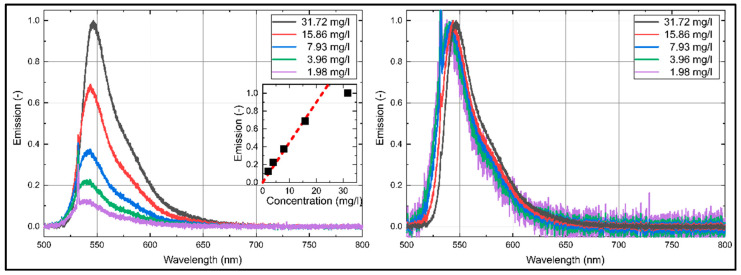
Emission spectra (**left**: normalized to maximum intensity of 31.72 mg/L; **right**: normalized to the respective maximum intensity) of Eosin-Y dissolved in water, inserted diagram show linearity (*R*^2^ = 0.993) of the integral LIF-signal for various dye concentrations; 293 K, 0.1 MPa.

The emission and absorption spectra of Eosin-Y dissolved in water at various dye concentrations at reference conditions (293 K, 0.1 MPa) are discussed in the following section. The respective absorption and emission spectra are shown in Figure 5 and Figure 6. The absorption spectra of Eosin-Y shows a distinct absorption between 430 nm and 560 nm. The absorption spectra are narrower compared to ethanol. They show one broad peak around 517 nm, which is slightly “blue-shifted” in comparison to ethanol. There is no significant variation in the absorption spectrum visible with changed dye concentration, see the spectra normalized to the individual maximum intensity in in Figure 5 (right). The absorption intensities increase linearly with higher dye concentration as well. The coefficient of determination *R*^2^ for the linear fit curve displayed in Figure 5 is 0.999. In contrast to ethanol, an increase in dye concentration shows no significant shift and change of the absorption spectra.

The emission spectra of Eosin-Y in water (Figure 6) show a distinct emission between 515 nm and 675 nm with one broad peak around 543 nm, which is again “blue-shifted” compared to ethanol. The emission intensities increase linearly with higher dye concentration up to 15.86 mg/L above which a saturation of the fluorescence is observed. The coefficient of determination *R*^2^ for the linear fit curve (up to 15.86 mg/L) displayed in is 0.993. With increasing dye concentration, the emission spectra are shifted slightly towards larger wavelengths, see the spectra normalized to the individual peak intensity in Figure 6 (right). The same behavior of the fluorescence of Eosin-Y dissolved in water was already reported by Koegl et al. in an earlier study [23] but for different dye concentrations.

### 3.2. Effect of the Laser Fluence on the Emission Spectra

**Figure 7:**
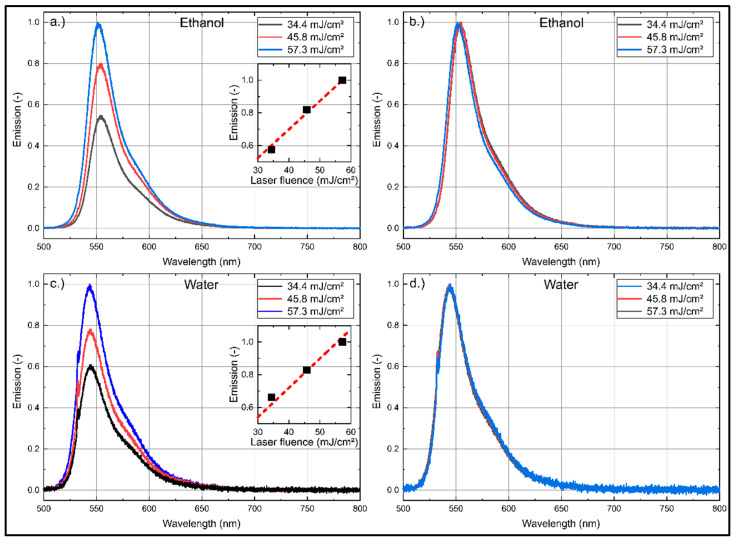
Emission spectra (left normalized to the respective maximum intensity; right: normalized intensity) of Eosin-Y dissolved in ethanol (**a**,**b**) and water (**c**,**d**), inserted diagrams show linearity (*R*^2^ = 0.999) of the integral LIF-signal for various laser fluences; 293 K, 0.1 MPa.

The influence of the laser fluence on the fluorescence of Eosin-Y (15.86 mg/L) dissolved in ethanol and water is shown in Figure 7. The emission intensities increase linearly with increasing laser fluence. The coefficient of determination *R*^2^ for the linear fit curves is 0.999 (for ethanol and water). An increase in laser fluence slightly shifts the emission spectra of Eosin-Y dissolved in ethanol towards lower wavelengths, which is negligible in terms of PDS. Eosin-Y dissolved in water shows no laser fluence dependent spectral shift at all.

### 3.3. Temperature Dependent Absorption and Emission Spectra

**Figure 8:**
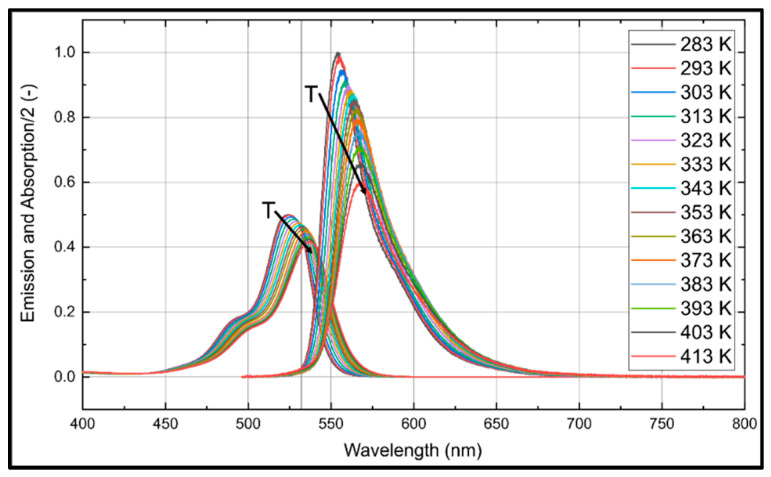
Normalized absorption (**left** curves) and emission spectra (**right**) for Eosin-Y (15.86 mg/L) dissolved in ethanol for various temperatures, 0.1 MPa (Please note that the absorption signal is divided by factor of 2 for clarity); the spectra are normalized to the maximum peak signal at 283 K.

**Figure 9:**
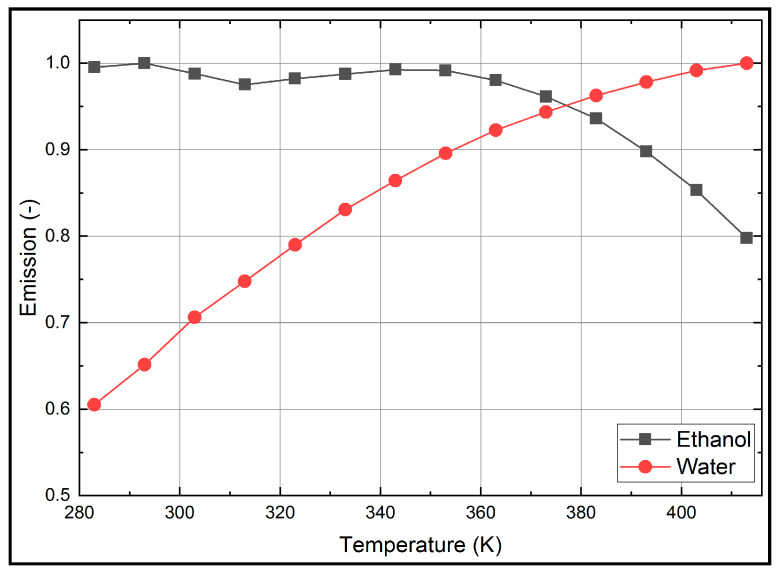
Normalized integral fluorescence intensities of Eosin-Y (15.86 mg/L) dissolved in ethanol and water for various temperatures, 0.1 MPa; the signals are normalized the respective maximum.

Droplet size measurements in spray systems are affected by evaporation-induced temperature changes. For a reliable determination of the droplet size, a temperature insensitive fuel–tracer system is mandatory. The temperature dependent absorption and emission spectra of Eosin-Y (for a dye concentration of 15.86 mg/L) dissolved in ethanol are shown in Figure 8 and the corresponding normalized integral fluorescence intensities are provided in Figure 9. The absorption spectrum is shifted steadily towards larger wavelengths. For example, at 283 K, the peak is positioned at 524 nm and at 413 K, it can be found at 537 nm. The absorption signal peak of Eosin-Y in ethanol decreases by 15.3% in the investigated temperature interval (283 K to 413 K).

The fluorescence intensity shows a slightly different behavior. The fluorescence spectra are shifted with increasing temperature towards higher wavelengths (from 554.0 nm to 568.6 nm). This spectral behavior is in agreement with the investigations reported by Mishra et al. [20] in the temperature interval from 298 K–338 K. The integrated fluorescence intensity decreases by 19.8% with increasing temperature within the investigated temperature range (283 K to 413 K), see Figure 9.

**Figure 10:**
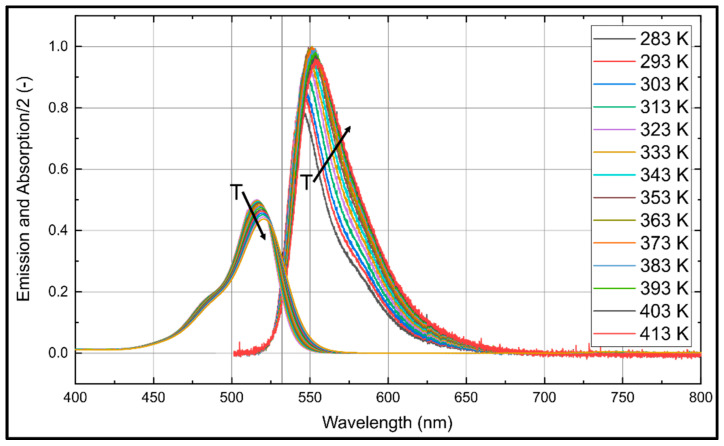
Normalized absorption (**left** curves) and emission spectra (**right**) for Eosin-Y (15.86 mg/L) dissolved in water for various temperatures, 0.1 MPa (Please note that the absorption signal is divided by factor of 2 for clarity); the spectra are normalized to the respective maximum peak signal at 283 K (absorption) or 393 K (fluorescence).

The temperature dependent absorption and emission spectra of Eosin-Y (15.86 mg/L) dissolved in water are presented in Figure 10 and the corresponding temperature dependent normalized integral fluorescence intensities are given in Figure 9.

The absorption signal of Eosin-Y in water at 283 K shows a peak at 516 nm, which is shifted uniformly 521 nm at 413 K. The absorption signal peak decreases uniformly by 12.5% within the investigated temperature interval (283 K to 413 K). The fluorescence intensity shows a slightly different behavior. The fluorescence intensity peak increases about 26.1% with increasing temperature within 283 K to 353 K. A temperature change from 353 K to 413 K leads to a slight decrease of the intensity, which is 19.9% higher in comparison to 283 K. The fluorescence spectra are shifted with increasing temperature towards higher wavelengths, i.e., between 544 nm (283 K) and 554 nm (413 K). These results are in agreement with previous investigations by Koegl et al. [23] (presented for a smaller interval of 293–333 K).

The normalized integral fluorescence (see Figure 9) for ethanol shows a minor temperature dependency up to 363 K. Although the peak signal drops with temperature up to 363 K, the spectra are broadened and shifted so that the integral signal is almost constant. Above this temperature, the normalized integral fluorescence signal shows a distinct temperature effect and the integral intensity decreases by 19.8%. The low temperature sensitivity of Eosin-Y dissolved in ethanol in the temperature range of 283 K to 363 K qualifies the suitability of the mixture for LIF/Mie droplet sizing. Temperatures above 363 K require a suitable temperature correction (e.g., by using a 2-color LIF approach) to compensate these effects and the related errors.

The normalized integral fluorescence behavior (see Figure 9) for water shows a significant temperature dependency for the completely investigated temperature interval. The normalized integrated emission increases by 65.2% from 283 K to 413 K. This behavior requires adequate temperature correction to ensure reliable PDS measurements.

### 3.4. Photo-Bleaching

**Figure 11:**
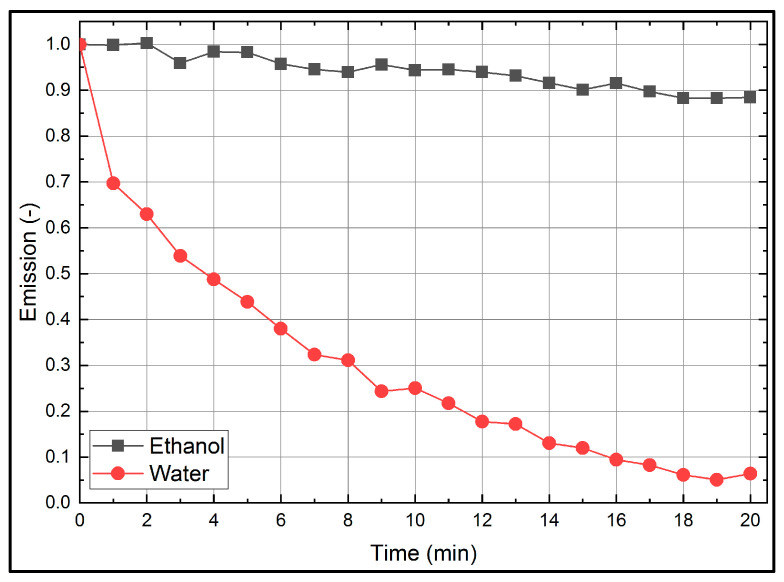
Photo-bleaching effect of Eosin-Y (15.86 mg/L) dissolved in ethanol and water at 293 K.

Finally, the effect of continuous illumination with constant laser fluence on the fluorescence signal is discussed. The so-called photo-dissociation of the dye and its effect on the fluorescence signal is discussed based on the normalized integral fluorescence intensities, which is displayed in Figure 11. The dye/fuel mixture was continuously illuminated (laser repetition rate 10 Hz, 45.8 mJ/cm^2^) for 20 min. The LIF-spectrum was measured every 60 s and the wavelength specific fluorescence intensities were summed up (The investigation showed a reduction (11.6% for ethanol; 93.6% for water) of the LIF-signal with time. The results indicate that multiple illumination cannot be neglected and have to be taken into account for the following investigations in droplets using the acoustic levitator. Due to its extremely high photo-dissociation, water is not suitable for long-time fluorescence measurements over several minutes in the levitator, since a few illuminations already lead to a significant reduction of the fluorescence signal.

## 4. Results of the Evaporating Droplet Studies

The following section deals with the droplet investigations in the acoustic levitator. Due to the significant photo-bleaching effect of water (see Figure 11), only fluorescence signals of ethanol droplets are investigated in the following section. The section is structured as follows: First, the evaporation rate of the droplet in the acoustic levitator is discussed and the evaporation dependent dye concentration is calculated from the droplet size. Effects of the droplet sphericity on the LIF-signals and droplet evaporation are discussed. Second, the fluorescence signal is analyzed for two different illumination frequencies in terms of photo-dissociation. For all measurements, 10 individual sequences (of single levitated droplets) are recorded each and are averaged.

**Figure 12:**
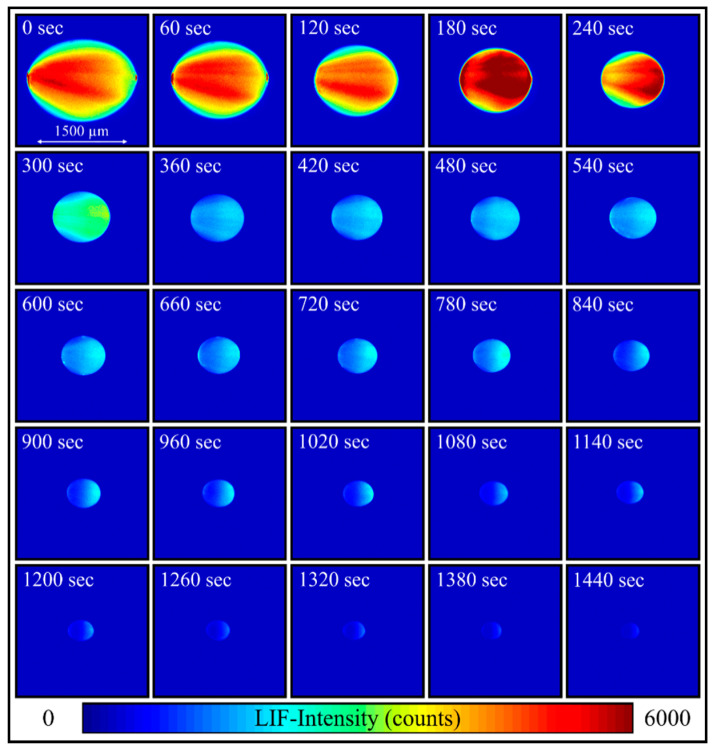
Sequence of a single evaporating droplet (initial temperature: 293 K), illumination frequency 1/60 Hz, the light sheet enters the droplet from the right).

The droplet evaporation is shown in Figure 12 based on a single LIF image sequence of an ethanol droplet (with an initial dye concentration of 15.86 mg/L). The laser light enters the droplet from the right side. The initial droplet size is about 1600 µm (sphericity at 0 s: 1.32; which is defined as the ratio of the ellipsoidal major axis *S_l_* and the ellipsoidal minor axis *S_s_*), which corresponds to a liquid volume of 3.5 µL. At first, the levitator’s acoustic waves squeeze the droplet, leading to an oblate shape. During evaporation, the influence of the surface tension increases and at 180 s, the droplet (with current diameter of ~900 µm) has a nearly spherical shape (sphericity: 1.14) and maintains this shape until the end of the measurement. The mean LIF signal intensity level of the droplet stays nearly constant within the first 120 s. At 180 s, the change from the oblate to a more spherical shape leads to an increase of the total fluorescence intensity due to changed focusing of the laser light and stronger internal reflections.

The LIF signal inside the droplet is larger in the right part, which is because of absorption and extinction effects within the droplet. Within the first 60 s, small glare points at the entrance and exit of the droplet are caused by Mie-scattering enhanced reabsorption effects. The Mie-scattering itself is filtered and is thus not visible in the LIF image. The intensity of the entrance glare point (which results due to reflection) is higher in comparison to the exting glare point (due to refraction). Afterwards, these glare points disappear probably due to extinction effects caused by the increasing dye concentration in combination with the low laser fluence. Morphology-dependent resonance (MDR) effects [5,30], which are common for pulsed laser operation, were not observed during the measurements, which is related to the low laser fluences applied. A lens effect due to the droplet interface leads to a focusing of the incidental laser beam [4]. This results in a non-uniform laser illumination and LIF-signal distribution inside the droplet. It is obvious that the LIF signal is not homogeneously produced in the whole droplet, which can explain deviations from the theoretical *d^3^*-dependency of the LIF signal [4]. These observations were also found for much smaller droplets [11]. Similar LIF-signal distributions in acetone-doped ethanol droplets were also found by Maqua et al. [31]. At increased acetone concentrations, light absorption by the dye was very strong in a very thin layer close to the surface, which reduces the local irradiance inside the droplet. Consequently, the increase of the dye concentration (dye enrichment) due to evaporation leads to a stronger laser light extinction and a decrease of the fluorescence intensity with time. The investigations in the previous section showed that a dye concentration above 15.86 mg/L in the cuvette (extinction length 10 mm) leads to a partial saturation of the fluorescence caused by absorption and extinction effects (both the laser light and the fluorescence are absorbed in the cuvette, see Figure 4). However, reabsorption of fluorescence towards the detector and LIF-signal drop is more distinct in the cuvette due to the long pathway.

**Figure 13:**
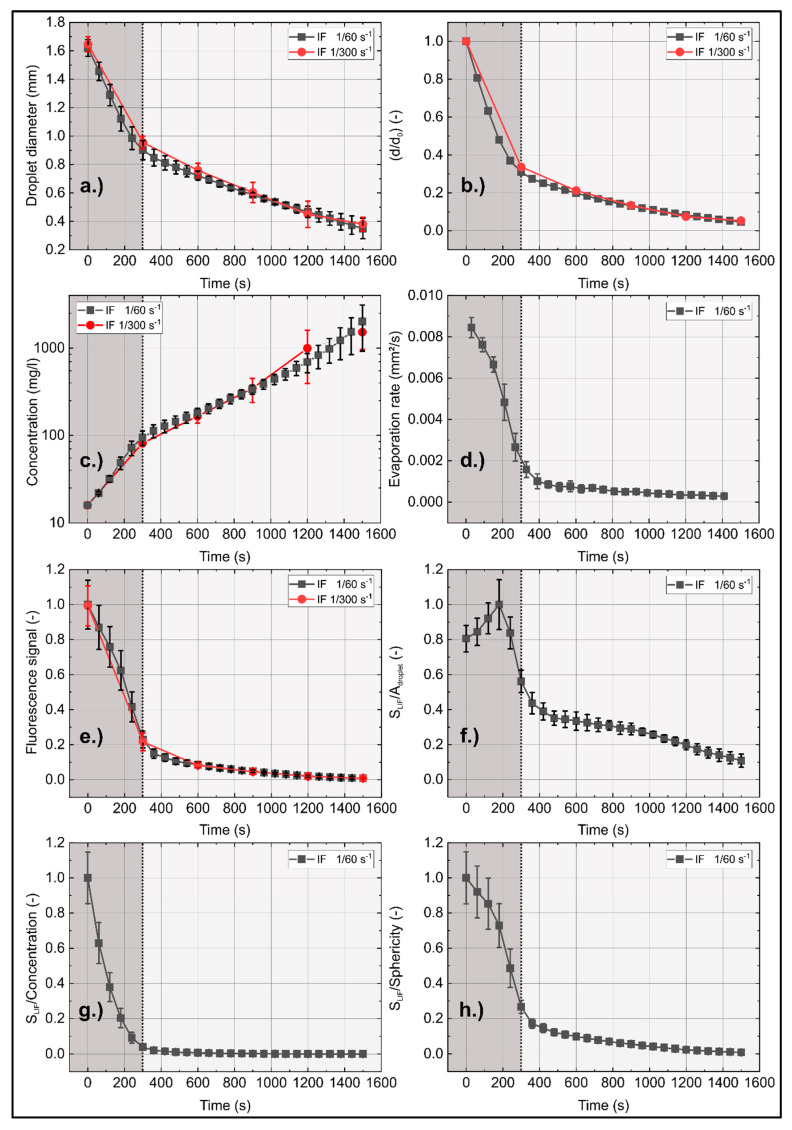
Time dependent averaged droplet diameter (**a**), (d/d_0_)^2^ behavior (**b**), dye concentration (**c**), evaporation rate ((**d**); d(d^2^)/dt), droplet fluorescence signal ((**e**), normalized droplet fluorescence signal to droplet area (**f**), normalized to concentration (**g**), and normalized to sphericity (**h**)), 293 K initial temperature. Two stage evaporation marked in gray (dark gray: first stage, light gray: second stage).

The droplet diameter is calculated in order to study the evaporation behavior. The droplet shape was assumed as rotational symmetric and the oblate shape was approximated by an ellipse. The volumetric equivalent diameter *d* was computed as follows [32]:

(1) $$d=\sqrt[{3}]{S_{s}S_{l}^{2}},$$

where *S_l_* and *S_s_* are the major and minor axis of the ellipse, respectively. In Figure 13, the two-stage evaporation of the droplet in terms of decreasing averaged droplet diameter with time, the variation of the droplet surface with time (in terms of the (*d*/*d*_0_)^2^ behavior; *d*_0_: initial droplet diameter, *d* actual droplet diameter), the change in dye concentration, the evaporation rate (d(*d*^2^)/d*t*), and the fluorescence signal are provided. Within the first stage (0 s–300 s, interval marked with dark gray overlay) the droplet rapidly decreases in size. After 300 s, the evaporation rate is reduced by a factor of 6 (interval marked with light gray). This change in evaporation rate is mainly caused by “acoustic streaming”, an effect which is well known [32,33,34,35]. In this case, a secondary gas flow around the droplet, which is stabilized by an acoustic field, is generated [32]. This flow is time-independent but is a function of the droplet shape. It is induced by the droplet, which acts as an obstacle in a periodic acoustic boundary-layer flow [33,35]. This secondary flow can be separated in an inner acoustic streaming field, which is directly located at the outer surface of the droplet. This inner field generates large-scale toroidal vortices, which form the outer acoustic streaming field [33]. This effect leads to an increase of the mass of vapor in the outer acoustic streaming field during evaporation and thus results in a reduced evaporation rate [32,33,34]. The influence of the acoustic streaming on the evaporation could be reduced by applying an external gas flow (either normal to the levitator axis [33]; or along the levitator axis [34,36,37]). Since the focus of the present study is not on the droplet evaporation in such special levitators, rather on the change in LIF-signal due to the shrinking droplet size, a basic acoustic levitator arrangement without an external gas flow is sufficient.

Due to the low laser illumination rates (1/60 s^−1^ or 1/300 s^−1^) and low laser fluences applied, an increase of the droplet temperature due to laser irradiance can be excluded. The temperature insensitive LIF signal of the fuel–dye mixture (see Figure 9) enables an investigation of the fluorescence of the evaporating droplet in a wide temperature interval (e.g., between 280 K and 360 K, see Figure 9) without a complex temperature correction (e.g., by using a 2-color thermometry concept). However, the fluorescence depends on several other quantities and boundary conditions.

The initial dye concentration is far below the concentration at which extinction and self-quenching becomes important (see Figure 6). The extinction length of the levitated droplet (initial extinction length *S_l_* = 1.6 mm) is much smaller in comparison to the cuvette (10 mm) used for the spectral measurements. The evaporation within the first 360 s leads to a dye concentration of 100 mg/L, which is 7-times higher than the initial concentration (see Figure 13c). The dye concentration increased by a factor of 126 after 1500 s, which leads to strong extinction effects and probably to re-absorption of fluorescence within the shrinking droplet. The evaporation rate (Figure 13d) shows the typical two-stage evaporation behavior attributed to acoustic streaming.

The course of the integral LIF intensity (Figure 13e) shows a complex behavior with reduced droplet size (for an illumination rate of 1/60 s^−1^). The fluorescence signal reduction of the evaporating droplet was already reported by Düwel et al. [38] (using the dye rhodamine 6G dissolved in water) for a droplet being fixed by two glass fibers. For a more detailed discussion, the area-, concentration-, and sphericity-normalized fluorescence intensities (see Figure 13f–h) are provided. A closer look at the integrated fluorescence intensity (*S*_LIF_) normalized to the droplet surface (A_droplet_) reveals that the normalized intensity initially increases up to 180 s and then finally decreases. This behavior is in agreement with the single droplet sequence in Figure 12, where the single droplet at 180 s shows the highest local intensities of the whole sequence. The sphericity is almost constant after 180 s (at about 1.14), while the evaporation rate still drops with similar slope until about 300 s. The integrated fluorescence intensity (*S*_LIF_) normalized to the sphericity (Figure 13h) shows a similar behavior as the integrated fluorescence signal (Figure 13e). The sphericity changes mainly within the first 180 s (it drops from 1.32 to 1.14, i.e., the droplet turns more spherical at 180 s), which leads to the different slope within this interval. After 300 s, the ratio of the integral LIF signal and the dye concentration is constant, see Figure 13g. This indicates that in this stage of evaporation the laser light extinction in the droplet is mainly responsible for the LIF signal intensity reduction, while it is not relevant in the very early period (i.e., 0 s–180 s, see also Figure 11).

In summary, the present study revealed that droplet deformation changes the illumination field inside the droplet, which may lead to lower integral signal intensities compared to droplets that are more spherical. Droplet evaporation leads to a dye enrichment in combination with laser extinction effects resulting in a proportional reduction if the integral fluorescence signal over time. Dye enrichment has a big impact on the uncertainty and has to be taken into account, especially when high ambient temperatures and/or long residence times are studied.

## 5. Conclusions and Future Work

The fluorescence of liquids doped with the dye Eosin-Y was studied at various conditions for utilization of LIF especially for droplet sizing in spray systems. First, the photo-physical properties of Eosin-Y in ethanol and water were analyzed in a micro cell under various conditions using a spectrometric LIF setup. The absorption and emission signals showed a linear trend with increasing dye concentration. A linear dependence of the fluorescence signal with increasing laser fluence and dye concentration was observed for the studied conditions. The absorption and emission signals were investigated in a wide temperature range (283 K–413 K). The absorption signal decreases with increasing temperature for both investigated liquid/dye mixtures and the spectrum is shifted towards higher wavelengths. The fluorescence signal shows a slightly different behavior. The fluorescence spectra of Eosin-Y dissolved in ethanol and water are shifted with increasing temperature towards higher wavelengths. However, the total fluorescence signal intensity of Eosin-Y dissolved in water shows a more distinct temperature dependence at moderate temperatures (283 K–363 K), while this is less significant in ethanol. Eosin-Y in water exhibits significant photo-dissociation effects under pulsed laser illumination, while ethanol only shows a slight reduction in intensity. This makes an investigation of water droplets with multiple Laser illuminations unfeasible.

In a second step, the individual LIF signals of the single droplets are studied with an acoustic levitator. The evaporation process shows a two-stage behavior. Within the first stage, the droplet rapidly decreases in size. Within these initial 300 s, the droplet size is reduced by the factor of 6. The fluorescence signal showed a complex behavior during evaporation. In principle, the integral LIF signal decreases with reduced droplet size. During evaporation, the dye concentration increased by a factor of 126, which leads to strong extinction effects within the shrinking droplet. The spherical shape of the droplet has a high impact on the emitted fluorescence signal as well. The lens effect caused by the droplet interface leads to a focusing of the incidental laser beam. For non-spherical droplets, a more inhomogeneous illumination field may result inside the droplet leading to a lower integral LIF signal. At later stages during evaporation (and for more spherical droplets), the laser extinction inside the droplet, due to higher dye concentrations, becomes the dominant effect reducing the integral LIF signal intensity.

Further extensive investigations have to be conducted for better understanding the effects of dye enrichment and droplet deformation on the resulting LIF signal. In particular, smaller droplets (<100 µm) must be studied for technically relevant spray systems. At higher ambient temperatures, thermal stratifications inside the droplet must be considered as well. These measurements can be used to increase the reliability of planar droplet sizing techniques based on LIF/Mie ratio.
